# Primary ovarian hydatid cyst mimicking cyst adenoma: a rare case report

**DOI:** 10.3205/dgkh000488

**Published:** 2024-06-05

**Authors:** Ensiyeh Bahadoran, Fatemeh Samieerad, Simindokht Molaverdikhani, Saeideh Gholamzadeh Khoei

**Affiliations:** 1 Student Research Committee, Qazvin University of Medical Sciences, Qazvin, Iran; 2 Department of Pathobiology, Faculty of Medical School, Qazvin University of Medical Sciences Qazvin, Kowsar Medical and Educational Center, Qazvin, Iran; 3 Kowsar Clinical Research Development Unit, Qazvin University of Medical Sciences, Qazvin, Iran; 4 Medical Microbiology Research Center, Qazvin University of Medical Sciences, Qazvin, Iran

**Keywords:** hydatid cyst, primary, ovary, echinococcosis

## Abstract

**Background:**

Hydatid cysts (HC) are zoonotic diseases that are mainly caused by *Echinococcus granulosus*. Ovarian HC is a rare condition with different and unspecified presentations. Here we report a rare case of primary ovarian HC.

**Case Presentation:**

A 47-year-old woman with chronic abdominal pain and left hemipelvic fullness was referred to the Obstetrics Clinic of the Kowsar Hospital of Qazvin. Abdominopelvic sonography revealed a cystic mass, which primarily suggested a cyst adenoma. The tumor marker levels were within normal limits. After surgical resection, histopathological examination showed a cystic mass with dimensions of 10×6×3 cm, smooth external and internal aspects, wall thickness of 0.3 cm, and multiple pieces of irregular gray membranous tissue. The patient was treated with albendazole 3 months after surgery, and a 6-month follow-up sonogram revealed no signs of recurrence.

**Discussion:**

HC has non-specific presentations. Radiologists, pathologists, and surgeons should consider HC as a differential diagnosis for any cystic mass in the pelvic cavity, especially in endemic areas. Surgical resection and albendazole administration are the chosen treatments.

## Zusammenfassung

**Hintergrund:**

Echinokokkuszysten (EZ) sind Zoonosen, die hauptsächlich durch *Echinococcus granulosus* verursacht werden. Echinokokkuszysten im Eierstock sind eine seltene Erkrankung mit unterschiedlichen, unspezifischen Präsentationen. In diesem Bericht stellen wir einen seltenen Fall einer primären Echinokokkuszyste im Eierstock vor.

**Kasuistik:**

Eine 47-jährige Frau mit chronischen Bauchschmerzen und einer gefüllten linken Becken wurde in die Geburtsklinik des Kowsar-Krankenhauses in Qazvin überwiesen. Die abdominale Sonographie zeigte eine zystische Masse, die zunächst auf ein Zystadenom hindeutete. Die Tumormarkerwerte lagen aber im normalen Bereich. Nach chirurgischer Resektion zeigte die histopathologische Untersuchung eine zystische außen und innen glatte Masse mit Abmessungen von 10×6×3 cm, einer Wanddicke von 0,3 cm und mehreren Stücken unregelmäßigen grauen membranösen Gewebes. Die Patientin erhielt 3 Monate nach der Operation eine Behandlung mit Albendazol; die 6-monatige Nachuntersuchung per Ultraschall ergab keine Anzeichen für ein Rezidiv.

**Schlussfolgerung:**

Echinokokkuszysten zeigen unspezifische Symptome. Radiologen, Pathologen und Chirurgen sollten EZ als differenzialdiagnostische Möglichkeit für jede zystische Masse im Beckenraum in Betracht ziehen, insbesondere in endemischen Gebieten. Chirurgische Resektion und die Verabreichung von Albendazol sind die bevorzugten Behandlungen.

## Introduction

Anthropozoonosis, known as hydatid illness, is caused by *Echinococcus (E.)* species tapeworms in the larval stages. *E. granulosus*, *E. multilocularis*, *E. oligarthrus*, and *E. vogeli* are the species involved in the disease, with *E. granulosus* being the most prevalent, accounting for 95% of cyst formations [1], [2]. In the intestines of carnivores, such as dogs (the parasite's definitive host), the worm attaches to the mucosa using hooklets. The eggs of the parasite are excreted in the feces of carnivores and are later ingested by herbivores like sheep and cattle, acting as the intermediate hosts for the parasite. Following this, the larva penetrates the intestinal wall and migrates throughout the body via the bloodstream [3], [4]. When the definitive host consumes the intermediate host's viscera, its life cycle is completed [5]. Humans can be incidental hosts [6][6], and can become infected by eating unwashed vegetables, drinking contaminated water, touching infected soil, or being near pet dogs [7].

Hydatid cysts (HC) are most frequently found in the liver and lungs [8]. Pulmonary HC grow more quickly than liver HC because the lungs have a softer consistency than the liver. Furthermore, because children’s lung tissues are more elastic than those of adults, HC in children grow larger and faster [9]. Approximately 0.2% to 2.25% of HC cases involve the ovary. It can appear in a primary or secondary form and shares morphological similarities with other areas. About twenty instances of primary ovarian HC have been documented [10]. The secondary form is more prevalent and is associated with multiorgan HC, lungs, or liver echinococcosis [10]. The pericyst, comprising the host’s inflammatory tissue, exocyst, and endocyst, where the scolecs and proligere membrane are generated, makes up the cyst's typical structure [5]. 

Based on its location, size, and host immunological response, this disease can exhibit different clinical presentations and complications. Symptoms can range from asymptomatic – due to the slow-growing nature of cysts in most cases – to anaphylactic shock due to cyst rupture or fistulization into adjacent organs [4], [11].

Ultrasound, computed tomography (CT), and magnetic resonance imaging (MRI) are the most frequently used imaging techniques for the diagnosis and follow-up of patients with HC. Ultrasound is used for HC staging and treatment planning [12], [13]. Based on the sonogram, cystic echinococcosis (CE) is divided into three classes according to the state of activity: active (CE1 and CE2) with clear contents and water-lily sign; transitional (CE3), with the immune system or drugs compromising the cyst; and inactive (CE4 and CE5), with calcification of the cyst wall [12]. Although ultrasound cannot provide a definitive answer, it is a viable option for screening, post-treatment monitoring, and cyst staging [14]. The cyst size, number, and local problems can all be observed on a CT scan, along with osseous organ involvement, and the presence of calcifications. In cases of biliary or neurological involvement, and to distinguish HC from neoplasms, MRI is preferred [4][4]. An enzyme-linked immunosorbent assay (ELISA) test in the active stages of the disease may be informative [15]. Pharmacological treatment, surgery, endoscopic interventional treatment, and subsequent minimally invasive techniques are some therapeutic approaches to hepatic hydatid disease [13].

Because HC is a rare entity, it should be considered in the differential diagnosis of any cystic mass in the pelvic cavity, especially in endemic areas, to provide optimal treatment before surgery and prevent accidental rupture of the cyst during surgery. In this paper, we present a rare case of an ovarian hydatid cyst in a 47-year-old woman and describe its management.

## Case presentation

Informed consent was obtained from the patient before enrollment in the study. A 47-year-old woman presenting with six months of chronic abdominal pain and left hemipelvic fullness without radiation was referred to the Obstetrics Clinic of Kowsar Hospital of Qazvin, Iran, in 2023. She had a history of three vaginal deliveries, normal menstrual cycles, no history of weight change or abdominal surgery, and no history of HC in herself or her family. She is a retired teacher who lives in the urban Qazvin. She had no contact with animals but occasionally traveled to rural areas. Physical examination of the abdomen revealed deep tenderness of the right suprapubic region with no palpable mass or skin abnormalities. No other abnormalities were detected on systemic or gynecological examinations.

**Figure 1:**
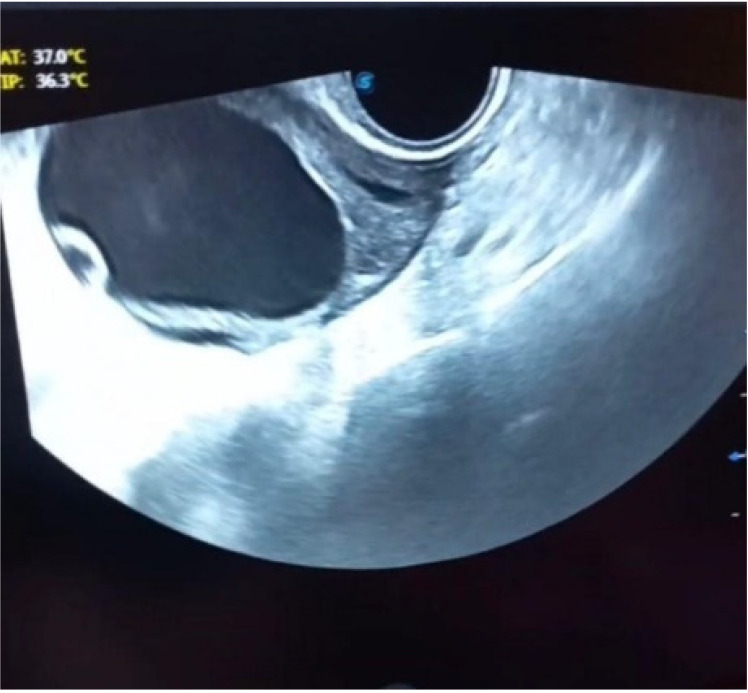
Cystic mass with a lobular margin shown by abdominopelvic sonography

Abdominopelvic sonography revealed a cystic mass with a lobular margin, and dimensions of 70×95×75 mm in the left hemipelvis, which lacked solid, nodulation, and calcification components, primarily suggesting a cyst adenoma. Chest imaging was normal (Figure 1 (Fig. 1)). The levels of tumor markers, including cancer antigen (CA)-125, carcinoembryonic antigen (CEA), alpha-fetoprotein (AFP), and beta-human chorionic gonadotropin (beta-hCG) were within normal limits. 

**Figure 2:**
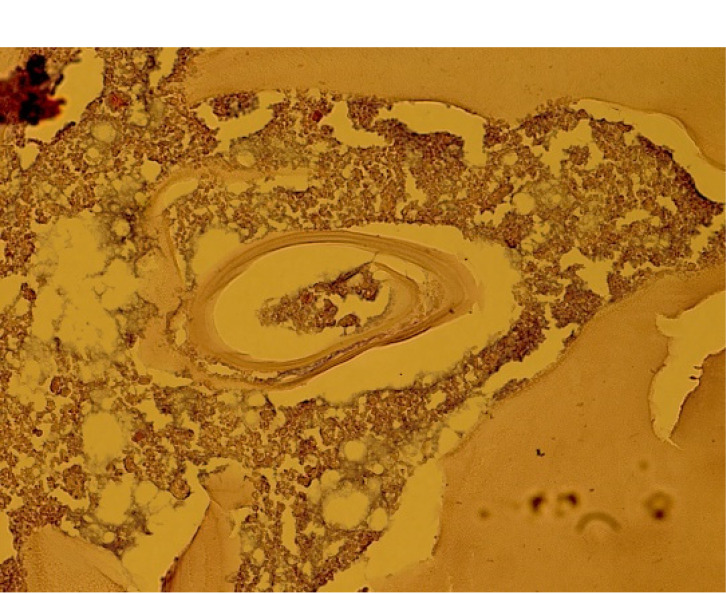
Ovarian hydatid cyst revealed fragmented acellular laminated layer, protoscolex admixed with necrotic material (400×, Hematoxylin & Eosin)

She underwent resection of the cystic lesion. On gross pathological examination, a cystic mass measuring 10×6×3 cm, with smooth external and internal aspects, wall thickness of 0.3 cm, and multiple pieces of irregular gray membranous tissue, were observed (Figure 2 (Fig. 2)). Albendazole treatment was initiated and continued for three months. The post-surgical course was uneventful and the follow-up sonogram showed no signs of recurrence after 6 months.

## Discussion

HC is a global parasitic ailment, prevalent in numerous regions where sheep and cattle are raised, with endemic occurrences. In various regions of North and East Africa, Europe, Asia, the Middle East, and South America, the illness is severely endemic [16]. Ovarian hydatid cysts are rare, with about two-thirds of cysts occurring as primary cysts; the majority of them have been reported in countries known as endemic zones, such as India, Iran, and Turkey [10]. Approximately four to five weeks after being infected with *E. granulosus* through the gastrointestinal route, larvae are released in the intestine. They then breach the epithelium, reaching the lamina propria and disseminating to other organs through lymphatic and blood circulation. They develop into hydatid cysts over the course of about five to fifteen years, eventually manifesting symptoms [11], [17]. HC are more prevalent in females than in males, as they have more contact with domestic animals and infected products [18]. Ovarian HC has been diagnosed at ages ranging from 12 to 76 years [10]. Consistent with this, our patient was a 47-year-old female, from an endemic area, who occasionally traveled to rural areas and was possibly infected through contaminated water or vegetables.

Involvement of pelvic organs is highly uncommon, given that the cyst often remains asymptomatic for an extended period before being diagnosed [3]. Symptoms typically arise in cases of cyst infections, upon cyst rupture, or due to the compression of adjacent organs or tissues by the cyst [19]. For ovarian HC, symptoms such as abdominal pain and distention [20], frequent urination [3] or urinary obstruction [21], pelvic pain or discomfort [22], postmenopausal metrorrhagia [23], occasional dysmenorrhea [24], and amenorrhea [25] have been reported. Our patient had hemi-pelvic fulness in addition to the most common symptom, i.e., abdominal pain.

Rupture can be triggered by trauma or may happen spontaneously because of elevated pressure in the cystic fluid. Key risk factors that make rupture more likely include a younger age, cyst diameter exceeding 10 cm, and the cyst being situated close to the surface [26]. In 16–25% of cases, rupture occurs, and in those situations, the rate of a severe reaction ranges from 1 to 12.5% [27]. It is possible to eliminate daughter cysts and lower the danger of allergic reactions by using solutions containing 0.5% cetrimide, 15% hypertonic saline, 1% silver nitrate, and sodium hypochlorite [28]. Cautious exposure and drainage have proven to be a safe and reliable treatment for peritoneal cysts strongly attached to intraperitoneal viscera. To avert secondary hydatidosis and allergic reactions, it is essential to isolate the abdominal cavity using gauze soaked in a 20% hypertonic saline solution [20].

Ultrasound, CT scan, MRI, and laboratory tests, are used to make the diagnosis. Only histological evidence can verify a final diagnosis [27]. The accuracy of serological testing can be affected by the size, location, and clinical phases of CE. The sensitivity of serological tests varies depending on the illness stage; for patients with inactive or early cystic stages, it is around 50%, while for those with active cysts, it is greater [29]. Our patient had one inactive cyst, with CE stages 4 and 5. A chest radiograph and abdominopelvic sonographic exam were performed to check for potential HCs in the liver or lung. In our case, neither the liver nor the lungs showed any signs of involvement.

In our case, the HC mimicked an ovarian cyst adenoma, which can also be asymptomatic, and when large, they present with abdominal and pelvic pain [30]. However, negative tumor markers ruled out this diagnosis. 

Surgery, either radical or conservative, is the most effective treatment for hydatid cysts, but it cannot prevent recurrence. Performing a complete resection is necessary to prevent the cyst from rupturing perioperatively. Treatment with benzimidazole compounds, such as albendazole or mebendazole, and the recently developed PAIR procedure (puncture-aspiration-injection-re-aspiration), which destroys the cyst’s germinal layer, offer additional treatment options for HC cases [31].

## Conclusions

Ovarian HC is a rare entity that occurs primarily without the involvement of the liver or lungs. This disease has different presentations, and non-specific radiological and laboratory findings. Radiologists, pathologists, and surgeons should be aware of this disease and consider it as a differential diagnosis for any cystic mass in the pelvic cavity, particularly in endemic areas. Surgical resection of the cyst is the gold standard of therapy, and with benzimidazole compounds before and after surgery, progression and recurrence can be eliminated.

## Notes

### Competing interests

The authors declare that they have no competing interests.

### Acknowledgment

We give our special thanks to the Clinical Research Center of Kowsar Hospital affiliated with Qazvin University of medical sciences.

### Authors’ ORCID

- Ensiyeh Bahadoran: 0000-0001-8299-1466
- Fatemeh Samieerad: 0000-0001-6091-4347
- Simindokht Molaverdikhani: 0000-0001-6602-0854
- Saeideh Gholamzadeh Khoei: 0000-0003-2675-9392
